# The effect of MemoVigor 2 on recent-onset idiopathic tinnitus: a randomized double-blind placebo-controlled clinical trial

**DOI:** 10.3389/fphar.2024.1252343

**Published:** 2024-01-24

**Authors:** Dimitrios G. Balatsouras, Isidora Papitsi, George Koukoutsis, Michael Katotomichelakis

**Affiliations:** ^1^ Department of Otorhinolaryngology, Tzaneio General Hospital, Piraeus, Greece; ^2^ Department of Otorhinolaryngology, Medical School, Democritus University of Thrace, Komotini, Greece

**Keywords:** tinnitus, antioxidants, placebo, oxidative stress, diet supplement, pharmacotherapy, clinical trials, hearing loss

## Abstract

**Background:** Tinnitus is a common symptom associated with the conscious perception of sound in the absence of a corresponding external or internal sound source, which can severely impact quality of life. Because of the current limited understanding of the precise pathophysiological mechanism of idiopathic tinnitus, no curable treatment has been attained yet. A food supplement trading as MemoVigor 2, which contains a combination of therapeutic ingredients with mainly antioxidant activity, has been used in the treatment of tinnitus. The objective of our study was to evaluate the effectiveness of MemoVigor 2 in the treatment of recent-onset idiopathic tinnitus.

**Methods:** In a prospective single-centre randomized, double-blind, placebo-controlled clinical trial we studied 204 patients with idiopathic tinnitus divided into two groups: 104 patients who received MemoVigor 2 and 100 patients treated with placebo. To evaluate changes in tinnitus we used (1) audiometry/tympanometry; (2) specific measures of tinnitus perception, including tinnitus pitch, loudness at tinnitus pitch, loudness at 1 kHz, minimum masking level, and residual inhibition; (3) questionnaires of tinnitus handicap inventory, mini tinnitus questionnaire and patients’ global impression of change. All patients underwent this test battery at the beginning of the study and in a repeat post-3-month session.

**Results:** All tinnitus measures, including pitch, loudness, minimum masking level and residual inhibition improved significantly in the intervention group. Most of these measures improved in the placebo group too, but in a lesser degree. All questionnaire scores diminished significantly in both groups, but the degree of decrease was greater in the intervention group. The participants’ tinnitus outcome reported after treatment using the patients’ global impression of change score differed significantly between the two groups, with greater improvement observed in the intervention group.

**Conclusion:** We found that the use of MemoVigor 2 improved recent-onset tinnitus, as proved by a set of tests performed for its evaluation, including audiometric measures, specific measures of tinnitus perception and tinnitus questionnaires. Tinnitus in the placebo group improved too, but to a lesser degree.

**Clinical Trial Registration**: isrctn.com, Identifier ISRCTN16025480

## Introduction

Tinnitus is the subjective perception of sound without the presence of a corresponding external or internal sound source. Most tinnitus cases are related to hearing impairment but nearly 25% of all patients with tinnitus have normal hearing thresholds (Langguth et al., 2019). There are two types of tinnitus, idiopathic or primary and secondary, with idiopathic tinnitus being by far the most frequent form (Tunkel et al., 2014). Idiopathic tinnitus is in most cases associated with sensorineural hearing loss and it is well-established that tinnitus often accompanies noise-induced hearing loss and presbycusis (Haider et al., 2018). Secondary tinnitus is caused by a specific underlying cause or an identifiable organic condition, which can be either an auditory disorder, such as cerumen impaction of the external auditory canal, middle ear disease, and various cochlear and auditory nerve abnormalities, or a nonauditory disorder, such as myoclonus, vascular anomalies, and intracranial hypertension.

Usually, the patients can cope well with their tinnitus, but nearly 10% experience bothersome tinnitus (Axelsson and Ringdahl, 1989) that can be accompanied by anxiety, depression, insomnia, stress, and emotional disturbance. Tinnitus prevalence ranges from 5.1% to 42.7% according to various reports and the prevalence of bothersome tinnitus ranges from 3.0% to 30.9% (McCormack et al., 2016). Its increasing frequency among the general population has become a major daily concern for all otolaryngologists.

The lack of knowledge about the precise pathophysiological mechanism of idiopathic tinnitus limits our ability to offer an effective treatment. Various therapeutic methods have been implemented on patients with tinnitus over time including educational counselling, medications, tinnitus retraining therapy, sound masking, biofeedback therapy, acupuncture, intra-tympanic injections and various other therapies with specific devices (Langguth et al., 2019)). However, a review of medical literature indicates that results are contradictory for most of the aforementioned therapies with the inevitable consequence that most patients cannot have their condition treated effectively (Tang D et al., 2019).

In recent years, a food supplement trading as MemoVigor 2 was introduced in the Greek market for the treatment of tinnitus. This medication is also in use at several European countries and contains a combination of therapeutic ingredients, including various vitamins, acetyl-L-carnitine, Ginkgo biloba (G. biloba) and Bilberry plant extracts, trace minerals and phospholipids. Most of them have antioxidant activity and have been previously used for the treatment of tinnitus, and also for the treatment of various peripheral and central vestibular syndromes, as well as for lesions of the acoustic and the facial nerve.

There are a few recent studies in which compounds of antioxidants have been also used for the treatment of idiopathic tinnitus. Petridou et al. (Petridou et al., 2019) treated 70 patients with idiopathic tinnitus, with a multivitamin-multimineral supplement including various vitamins, phytochemicals, minerals and a-lipoic acid, in a randomized, double-blind, placebo-controlled clinical trial. The authors found tinnitus improvement in the antioxidant group in the tinnitus questionnaires and the specific measures of tinnitus perception used and concluded that antioxidant therapy seems to reduce the subjective discomfort and tinnitus intensity in tinnitus patients. More recently, Knäpper et al. (Knäpper et al., 2023) studied the effectiveness of Tinnitan Duo^®^, a food supplement containing G. biloba, 5-hydroxytryptophan, magnesium, melatonin, vitamins B5 and B6, and zinc at improving tinnitus. The authors studied 29 patients who completed the study from a total of 61 patients and found that the food supplement was associated a significant improvement in tinnitus according to THI and tinnitus loudness measurements. The main limitations of this study were the small sample and the absence of a placebo group.

The objective of the present study was to evaluate the effectiveness of the MemoVigor 2 food supplement in the treatment of recent-onset idiopathic tinnitus.

## Materials and methods

**FIGURE 1:**
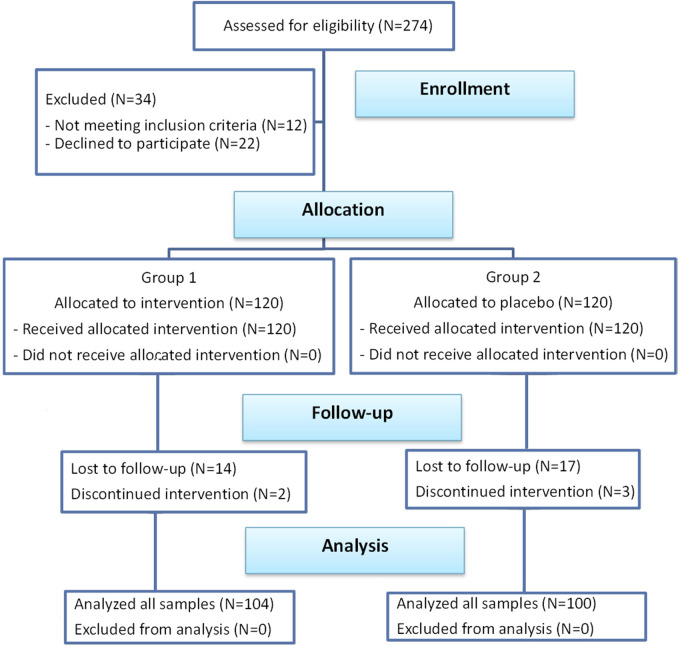
Schematic flow diagram of the clinical trial according to CONSORT guidelines.

This was a prospective single-centre randomized, double-blind, placebo-controlled clinical trial reviewed and approved by the Institutional Review Board of Tzaneio General Hospital of Piraeus (Ref.14549-49-05/12/2019). The research was conducted in accordance with the principles of the Helsinki Declaration of 1975 as revised in 2013 and was registered with ISRCTN (reference: ISRCTN16025480). The clinical trial of this study followed the guidelines of CONSORT (Figure 1).

The primary outcome measure was tinnitus change measured using the Tinnitus Handicap Inventory questionnaire (THI) at baseline and post-3-month. The secondary outcome measures were tinnitus severity measured using Mini Tinnitus Questionnaire (Mini-TQ) at baseline and post-3-month and tinnitometry findings including tinnitus pitch matching, tinnitus loudness matching, tinnitus minimum masking level (MML) and residual inhibition (RI) of tinnitus measured at baseline and post-3-month. Also, tinnitus perception change was estimated from the patients at the end of the study using the Patients’ Global Impression of Change (PGIC). Additionally, evaluation of hearing was also performed using audiometry at baseline and post-3-month.

### Inclusion-exclusion criteria

The study was performed at the Audiology-Neurotology Department of the tertiary referral setting of Tzaneio General Hospital of Piraeus, Greece. Patients who presented with tinnitus were enrolled based on the following inclusion criteria: (1) Both males and females, aged between 18 and 70 years; (2) Unilateral or bilateral idiopathic tinnitus of recent onset ≤12 months; (3) Patients had not received any previous medical treatment or had undergone treatment without success. Since in most cases, idiopathic tinnitus is associated with sensorineural hearing loss, including presbycusis and noise-related hearing loss (Dalrymple et al., 2021), patients with these clinical entities were included in the study. Participant exclusion criteria were: !1) Otosclerosis; (2) Chronic otitis media; (3) Meniere’s disease; (4) Hypo- or hyperthyroidism; (5) Diabetes mellitus; (6) Uncontrolled hypertension; (7) Hypercholesterolemia; (8) Coagulation disorders; (9) Use of anticoagulants, ototoxic or potentially tinnitus-inducing medications; (10) Active malignant diseases; (11) Autoimmune diseases; (12) Cardiovascular, renal or hepatic disorders; (13) Psychiatric disorders; (14). Somatosensory tinnitus, either alone or concurrrently with idiopathic tinnitus. Diagnosis of somatosensory tinnitus was obtained using the Sanchez and Rocha set of diagnostic criteria (Sanchez and Rocha, 2011), as revised by Michiels et al. (Michiels et al., 2018).

### Power analysis and sample size estimation

A repeated measures power analysis was conducted for the sample size calculation. Power analysis methodology represented a design, with two groups of the between-subject factor and two levels of the within-subjects factor of time for the primary outcome. For this design, 100 participants per group achieved a power of 0.95 for the between-subjects main effect at an effect size of 0.25; a power of 0.99 for the within-subjects main effect at an effect size of 0.20; and a power of 0.99 for the interaction effect at an effect size of 0.20. The sample size was initially increased to 120 participants per group in case some patients were lost to follow up.

### Randomization and blinding process

All consecutive patients with subjective tinnitus examined in our |Clinic, who fulfilled the inclusion criteria, participated in the study. The investigators informed the eligible patients regarding the aims, methods, anticipated benefits and potential hazards of the study, and provided them with an information sheet. Informed consent was obtained from each patient who agreed to participate in the study. Subjects were randomly allocated to take either one tablet of 900 mg of MemoVigor 2, or a placebo tablet daily for 3 months, in a 1: 1 ratio. MemoVigor 2 and placebo tablets were identical in colour, weight and size, and were both provided by the manufacturing company of MemoVigor 2, EUROPHARMA, placed inside bottles with identical appearance, with each bottle containing tablets for a 3-month treatment. A completely randomized design was used for both treatments using the Number Generator of the Research Randomizer (www.randomizer.org). Two sets of numbers were created and an independent contributor not involved in the study, assigned each number to one bottle. The independent contributor gave each patient a bottle, recorded the randomization number corresponding to each patient and created a list matching drug numbers with treatments, which was unavailable to investigators. Only the independent contributor had access to the group assignments and neither the patients nor the investigators were aware of the treatment allocation.

### Intervention

Randomized patients were given either one tablet 900 mg of MemoVigor 2 (EUROPHARMA, Torino, Italy) or a placebo, daily. MemoVigor 2 is a commercial product with batch number EUPH 0034 M10550_11/23 that contains various ingredients, such as phospholipids, L-acetylcarnitine, vitamins B1, B6, B12, C, and E, Ginkgo biloba and Bilberry plant extracts, as well as trace minerals, including selenium, magnesium and potassium. The full list of ingredients with the amount per tablet is shown in Table 1. Ginkgo biloba extract is 3% dry extract prepared from the leaves of the plant *Ginkgo biloba L.* [Ginkgoaceae], extraction solvent: ethanol and excipient: maltodextrin 35%. It contains ≥3% flavonol glycosides and ≤ 5ppm ginkgolic acids and is prepared from Suisse Nutraingredients, Monza, Italy. Bilberry extract is 1% dry extract prepared from the fruits of the plant Vaccinium myrtillus L. [Ericaceae], extraction solvent: water and excipient: maltodextrin 35%. It contains ≥1% anthocyanins and is prepared from Fagron Italia, Bologna, Italy. The intervention lasted 3 months and the patients were instructed to continue their usual medical treatment, diet, and exercise habits during this period and were advised to record and report any adverse reaction. Patients were recruited between December 2019 and March 2023.

**TABLE 1:** Composition of MemoVigor 2.

Ingredients	mg/tablet
Vitamin B1	0.825
Vitamin B6	1.05
Vitamin B12	0.00125
Vitamin C	40.00
Vitamin E	6.00
Ginkgo biloba 3% dry extract	50.00
Bilberry 1% dry extract	50.00
Acetyl-L-Carnitine hydrochloride	250.00
Linoleic acid with calcium salt	19.00
Magnesium gluconate	20.00
Potassium gluconate	50.00
Selenium	0.023
Phospholipid complex	95.00
- phosphatidylserine 22%	20.90
- phosphatidylethanolamine 16%	15.20
- phosphatidylcholine 15%	14.25
- phosphatidylinositol 10%	9.50
L-Glutamic acid	90.00

### Initial session

#### Medical history

The initial evaluation of the patients included taking a detailed medical history from every subject, and recording basic epidemiological data and the following: (1) Presence of hearing loss, vertigo or feeling of pressure in the ear; (2) Otological history; (3) Neurological symptoms; (4) Psychological problems (anxiety, depression, insomnia); (5) Presence of systemic diseases; (6) Drug intake; (7) History of noise exposure. Regarding tinnitus, the following data was recorded: (1) Months since tinnitus onset; (2) Laterality (right ear, left ear, bilateral, unspecified); (3) Description (whistling, ringing, blowing, roaring, etc.); (4) Type (pulsatile, respiratory synchronous, continuous or intermittent, steady or changing; (5) Intensity (mild, moderate, severe).

#### Clinical examination and laboratory evaluation

All patients underwent a clinical otolaryngologic and screening neurologic evaluation. Laboratory testing included blood cell count with erythrocyte sedimentation rate, and biochemical tests (total cholesterol and triglycerides, blood sugar, electrolytes). In selected cases, thyroid blood tests were performed as well. Moreover, in cases suspected of possible retrocochlear involvement, magnetic resonance imaging (MRI) of the internal auditory canal/cerebellopontine angle was ordered.

#### Audiometric and tympanometric evaluation

Audiological examinations were carried out in a soundproof booth. Pure-tone audiometry was performed using a GSI 61 Clinical Audiometer (Grason Stadler, Madison, United States) and standard TDH-49 headphones. Μeasurements were made with an ascending-descending technique (International Organization for Standard Acoustics, 1989), in 5 dB steps and all thresholds were calculated in dB HL at the frequencies of 0.25, 0.5, 1, 2, 4, 6 and 8 kHz. A maximum hearing threshold of 120 dB was recorded, and hearing thresholds exceeding this value were treated as a 120 dB loss. Standard single-frequency tympanometry was also performed by an impedance audiometer (TM 262 Autotymp Tympanometer, Welch Allyn, New York), using an 85 dB sound pressure level tone set at 226 Hz. In case of abnormal tympanometric results due to middle ear disease, the patients were rescheduled for evaluation after appropriate treatment, and when results remained abnormal, they were excluded from further study.

#### Specific measures of tinnitus perception

The multidisciplinary European guideline for diagnostics, assessment, and treatment of tinnitus recommends evaluation of the perceptional quality of tinnitus (loudness, pitch, and minimum masking estimations), as part of the minimum patient assessment (Cima et al., 2019). Some authors find psychoacoustic tinnitus measures controversial and probably with small relation to the impact of tinnitus (Manning et al., 2019). However, most authors suggest that this method is an important tool in the characterization of tinnitus and documentation of the efficacy of the adopted therapy (Raj-Koziak et al., 2019; Jin and Tyler, 2022). Accordingly, we used the following tests:

a. Tinnitus pitch test. Perceived tinnitus pitch can be a broad spectrum or a single tone. The tinnitus pitch test attempts to identify a pitch similar to the patient’s prominent sound or the central frequency of the perceived sound spectrum. We used the two-alternative forced choice applied to the ipsilateral ear. Two pulsed tones with different octave frequencies are presented alternately and the patients are instructed to judge whether the pitch of the first or the second tone is closer to their tinnitus. The examiner brackets with successive approximations until the desired accuracy is obtained. We repeated the tinnitus pitch test 5-7 times to increase its reliability and we used the average value in the analysis of the results.
b. Tinnitus loudness test. Tinnitus loudness was measured both at the participant’s tinnitus pitch and at 1 kHz. The external sound is presented and is increased or decreased in intensity until the patients judge that the loudness is equal to that of their tinnitus.
c. MML. The MML refers to the minimum level of broadband noise or octave noise required to make a patient’s tinnitus inaudible. We used a 2–12 kHz broadband noise as the sound stimulus and performed the test monaurally in the affected ear in cases with unilateral involvement or binaurally in cases with bilateral tinnitus. The stimulus is presented initially at low levels and using an ascending method, the intensity is increased in 2 dB increments to find a level at which tinnitus is totally covered. The test is repeated 2-3 times and if the test results are within 5 dB, the average is recorded as the final MML result.
d. RI. The phenomenon of short-term tinnitus suppression by different forms of acoustic stimulation is referred to as RI. After the masker is turned off, tinnitus may return to normal immediately, may be diminished or may disappear for some time and then gradually return to normal. We tested RI binaurally at the intensity level of 10 dB above the MML value presenting a white noise stimulus for 60 s. Subjects are instructed to listen to the noise during this period and then assess whether the tinnitus is gone, diminished or unchanged. If the tinnitus is completely absent after the 60-s exposure to the masking noise, it is referred as complete RI (C). If the tinnitus is reduced but not completely absent it is referred as partial RI (P). Finally, if the tinnitus remains unchanged, it is referred as absent RI (A).

#### Questionnaires

The overall impact of tinnitus is influenced by the perceptional quality of tinnitus which can be measured using the previously mentioned tests and by the individual’s psychological reaction to tinnitus, which can be evaluated using specific tinnitus questionnaires. Several questionnaires have been tested for validity and reliability (Jin and Tyler, 2022) to assess the severity of tinnitus and to evaluate the effects of tinnitus treatment. We used the THI and the Mini-TQ, both in the Greek language (Panagiotopoulos et al., 2015; Papitsi et al., 2020), and to assess the patients’ overall estimation of the outcome of treatment we used the PGIC score.

a. THI. The THI is a 25-item self-report questionnaire, first introduced by Newman et al., in 1996. The THI consists of a total of 25 questions which are further categorized into three subscales: functional, physical and catastrophic. Three responses are available for each question: Yes = 4 points; Sometimes = 2 points; No = 0 points. The total score ranges from zero to 100, with higher scores indicating greater perceived handicap. Guidelines for the classification of tinnitus severity constitute no handicap (0–16), mild handicap (18–36), moderate handicap (38–56), or severe handicap (58–100).
b. Mini-TQ. Mini-TQ is the abridged version of the widely accepted reliable Tinnitus Questionnaire (Hallam et al., 1988), which aims to assess the psychological effects of tinnitus-related distress and to investigate the dimensions of the complaint about tinnitus. Mini-TQ provides an equally reliable but far more rapid measure of tinnitus-induced distress (Hiller and Goebel, 2004). It consists of 12 items regarding tinnitus symptoms. Patients indicate the frequency/intensity of these symptoms with the use of a 3-point ordinal Likert Scale ranging from 0 to 2: True = 2 points; partly true = 1 point; not true = 0 points.
c. Patients’ Global Impression of Change (PGIC). On the PGIC (Hurst and Bolton, 2004), participants reported their perception of tinnitus change due to intervention, if any. Participants chose from 5 options: 0, no change or worsened; 1, small improvement; 2, moderate improvement; 3, significant improvement; 4, remission.

### Repeat session

During the post-3-month session, the clinical examination as well as the audiological and specific tinnitus measurement tests were performed again. All patients were asked to complete the THI questionnaire and the Mini-TQ again and to provide the PGIC score.

### Statistical analysis

Research data was imported into the statistical computer program SPSS, Release Version 26.0 (SPSS, Inc., Chicago IL, United States) for further evaluation and analysis. Continuous variables are presented with mean and standard deviation (SD) and quantitative variables are presented with absolute and relative frequencies. For the comparison of proportions, chi-square and Fisher’s exact tests were used. For the comparison of study variables between the intervention group and the placebo group, the Student’s t-test was computed.

Ηearing thresholds were analyzed using repeated measures analysis of variance (RANOVA). Two separate three-way RANOVAs of hearing thresholds were performed for the subjects of the intervention group and for the subjects of the placebo group. The within-group measured factor for RANOVAs was Frequency separated by the between-group factors Ears (Right/Left) and Session (initial and final: post-3-month). The dependent variable Frequency was measured across the frequency bands centered at 0.25, 0.5, I, 2, 4, 6, and 8 kHz (repeated measures factor Frequency with seven levels). All double and triple interaction terms were checked. Comparison of the hearing thresholds between the two groups was performed using mixed-model analysis of variance (ANOVA), for each session separately. In this analysis, Ear (Right/Left) and Frequency (0.25, 0.5, 1, 2, 4, 6 and 8 kHz) were the within variables and Group (intervention and placebo group) was the between factor. All double and triple interaction terms were checked. To compensate for the violations of sphericity in all ANOVAs and compound symmetry for within-groups factors, the Greenhouse and Geisser approach was adopted.

Values of tinnitus pitch, loudness at dominant frequency, loudness at 1 kHz, MML, THI and Mini-TQ were compared between the study and the control group with the Mann-Whitney test. Additionally, a comparison was made between two subgroups with tinnitus duration of 1-6 months and 7-12 months, in both, the study and the control group, to estimate a possible effect of spontaneous remission of tinnitus, which is more common during the first 6 months. Also, time comparisons were examined in the aforementioned values within each group, with the Wilcoxon sign test. The degree of change of these values throughout the follow-up period was compared between the two groups, with repeated measures ANOVA after having them logarithmically transformed. RI was compared between the two groups with Pearson’s chi-square test and between time measurements with the McNemar test. Tinnitus change was compared between the two groups with Pearson’s chi-square test. Again, the two subgroups with tinnitus duration 1-6 months and 7-12 months were compared, for both the study and the control group. All reported *p* values are two-tailed, with statistical significance set at *p* < 0.05.

## Results

Two hundred and seventy-four patients were enrolled in the study. Twelve patients did not fulfil the inclusion criteria and 22 patients refused to participate. Out of the 240 remaining patients, 120 were allocated to the intervention group and received MemoVigor 2 and 120 patients were allocated to the placebo group. Patients were recruited in one investigational centre from December 2019 to March 2023. All subjects were white/caucasian. Fourteen patients from the intervention group and 17 patients from the placebo group missed to follow-up mainly owed to hospital avoidance because of the Covid pandemic and were excluded from further study. Also, 2 and 3 patients from the two groups respectively discontinued intervention for reasons irrelevant to the study. Accordingly, 104 patients from the intervention group and 100 patients from the placebo group were available for our research (Figure 1). Patient compliance was good. The average missed tablets in the intervention group were three and in the placebo group were four. No adverse reactions were mentioned in either of the two groups.

Both treatment groups were similar concerning demographics and baseline characteristics presented in Table 2. The mean age was 57.7 years (SD = 11.7) for the intervention group and 56.3 years (SD = 11.5) for the placebo group. Hearing loss, history of noise exposure, tinnitus laterality, continuity, stability, description and severity were similar in the intervention and the placebo group. In 10 patients from the intervention group and 12 patients of the placebo group MRI of the internal auditory canal/cerebellopontine angle was performed with negative results.

**TABLE 2:** Intervention Group and Placebo Group demographics and baseline characteristics.

	Intervention group	Placebo group	*p*-value
Sex			
Male, N (%)	54 (51.9)	51 (51.0)	0.895^a^
Female, N (%)	50 (48.1)	49 (49.0)	
Age in years (mean ± SD)	57.7 ± 11.7	56.3 ± 11.5	0.896^c^
Ears with hearing loss, N (%)			
Normal or near-normal (HL ≤ 25 dB)	117 (56.2)	119 (59.5)	0.408^b^
Mild (HL 26-40 dB)	62 (29.8)	60 (30.0)	
Moderate (HL 41-55 dB)	22 (10.6)	19 (9.5)	
Moderately severe (HL 56-70 dB)	7 (3.4)	2 (1.0)	
Severe or profound (HL ≥ 71 dB)	00	00	
Vertigo, N (%)	8 (7.7)	6 (6.0)	0.783^b^
Ear pressure, N (%)	4 (3.8)	4 (4.0)	1.000^b^
Medications, N (%)			
Antidepressants	4 (3.8)	4 (4.0)	0.840^b^
Anxiolytics-Sedatives	3 (2.9 (	4 (4.0)	
Hypnotics	1 (0.9)	2 (2.0)	
Antiepileptics	1 (0.9)	0	
Diseases, N (%)			
Depression	4 (3.8)	5 (5.0)	0.878^b^
Stress	7 (6.7)	5 (5.0)	
Migraine	5 (4.8)	3 (3.0)	
Panic attacks	2 (1.9)	2 (2.0)	
Noise exposure, N (%)	12 (11.5)	13 (13.0)	0.750 =
Duration of tinnitus in months (mean ± SD)	8.7 (3.7)	8.1 (3.3)	0.186^c^
Tinnitus laterality, N (%)			
Right ear	26 (25.0)	19 (19.0)	0.722^b^
Left ear	22 (21.2)	20 (20.0)	
Both ears	52 (50.0)	56 (56.0)	
Unspecified	4 (3.8)	5 (5.0)	
Tinnitus continuity, N (%)			
Continuous	62 (59.6)	59 (59.0)	0.928^a^
Intermittent	42 (40.4)	41 (41.0)	
Tinnitus stability, N (%)			
Stable	82 (78.8)	80 (80.0)	0.838^a^
Changing loudness	22 (21.2)	20 (20.0)	
Tinnitus description, N (%)			
Whistling	87 (83.6)	88 (88.0)	0.298^b^
Blowing	10 (9.7)	10 (10.0)	
Ringing	7 (6.7)	2 (2.0)	
Tinnitus severity, N (%)			
Mild	72 (69.2)	63 (63.0)	0.224^a^
Moderate	18 (17.3)	27 (27.0)	
Severe	14 (13.5)	10 (10.0)	

^a^ Pearson’s chi square.

^b^ Fisher’s exact test.

^c^ Student’s t-test.

### Audiometry-tympanometry

**FIGURE 2:**
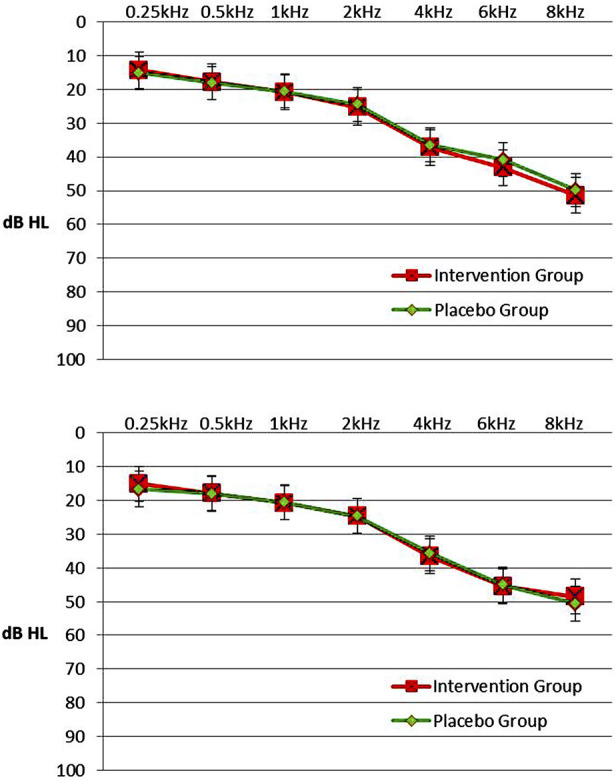
Upper panel. Mean hearing thresholds with standard errors in the frequency range from 0.25 to 8 kHz (dB HL) in the intervention group and the placebo group in initial session. 2. Lower panel. Mean hearing thresholds with standard errors in the frequency range from 0.25 to 8 kHz (dB HL) in the intervention group and the placebo group in post-3-month session. Left and right ears were pooled together since there was not any statistical difference in hearing thresholds between ears.

Tympanograms were normal (type A), on initial evaluation and on final evaluation, for both subjects and controls. The RANOVA conducted on the hearing thresholds of the intervention group showed that only the main effect of Frequency was statistically significant (F = 579.34, *p* < 0.001). Thus, mean hearing thresholds increased significantly as frequency increased. Also, the Ear’s main effect was not significant (F = 0.002, *p* = 0.869) indicating that no significant overall differences were found in hearing thresholds between left and right ear. The Session’s main effect was not significant as well (F = 0.001, *p* = 0.958), indicating the absence of significant deterioration in hearing, probably owed to the small time period elapsed between the two sessions. As far as the double interactions and triple interaction were concerned, it was found that all interactions were statistically nonsignificant. In the placebo group, only the main effect of Frequency was statistically significant (F = 614.05, *p* < 0.001). Thus, mean hearing thresholds increased significantly as frequency increased. Also, Ear’s main effect was not significant (F = 0.16, *p* = 0.687) and Session’s main effect was not significant (F = 0.03, *p* = 0.847). All double interactions and the triple interaction were statistically non significant. The effect of Frequency was significant as expected on both groups because higher frequencies gradually deteriorate in comparison with the middle and lower frequencies, owing to a certain degree of presbycusis observed in many patients or other causes of sensorineural hearing loss. Figure 2 (upper panel) shows a plot of mean hearing thresholds for the intervention group’s and the placebo group’s ears in the initial session. In this figure it is evident that mean hearing thresholds are higher in the high frequencies, a finding consistent with the RANOVA outcome.

A comparison of hearing thresholds between groups was conducted with a mixed model ANOVA, separately in each session. In the initial session, only Frequency’s main effect was significant (F = 415.56; *p* < 0.001). Ear’s main effect was not significant (F = 0.09, *p* = 0.763), as well as the Groups’ main effect (F = 0.11; *p* = 0.738). All double interactions and triple interaction were not significant.

In the post-3-month session, the Frequency’s main effect was significant again (F = 405.47, *p* < 0.001). Ear’s main effect and Group’s main effect were not significant (F = 0.58, *p* = 0.449 and F = 0.03, *p* = 0.859, respectively). All double interactions and triple interaction were also not significant. Overall, the mixed-model ANOVAs for comparison of hearing thresholds between the intervention group and the placebo group showed the absence of statistical differences between the two groups in both sessions. Mean hearing thresholds and SD in patients of the intervention and the placebo group, by Frequency, Ear and Session are presented in Table 3. Figure 2 (lower panel) shows a plot of mean hearing thresholds and SD for the ears of the intervention group and the placebo group, obtained in the post-3-month session. In this figure it is evident that mean hearing thresholds are again higher in the high frequencies, a finding consistent with the RANOVA outcome. Also, the comparison between the two panels shows similar hearing threshold results in both sessions.

**TABLE 3:** Mean hearing threshold in dB HL and standard deviation (SD) in Intervention Group and Placebo Group, by Frequency, Ear and Session.

Group	Frequency (kHz)	Right ear		Left ear	
		Pre	Post	Pre	Post
		Mean (SD)	Mean (SD)	Mean (SD)	Mean (SD)
Intervention group (N = 104)	0.25	14.4 (13.3)	15.0 (11.6)	14.3 (14.0)	15.3 (13.3)
	0.5	18.2 (12.9)	17.8 (13.2)	17.3 (14.4)	18.0 (13.5)
	1	20.7 (14.3)	20.7 (13.9)	20.8 (14.2)	20.7 (14.3)
	2	25.2 (17.2)	23.8 (17.0)	25.2 (16.7)	25.7 (16.9)
	4	37.1 (20.6)	36.6 (19.70)	37.3 (21.6)	36.8 (21.4)
	6	43.1 (19.9)	45.0 (20.10)	43.2 (22.1)	45.9 (26.7)
	8	51.5 (25.0)	48.1 (24.8)	51.2 (25.8)	49.0 (26.9)
Placebo group (N = 100)	0.25	14.5 (8.8)	16.4 (8.5)	15.8 (10.7)	17 (10.4)
	0.5	18.1 (10)	17.8 (8.4)	18.1 (9.4)	18.5 (16.5)
	1	21 (11.5)	20.8 (11.2)	20.3 (12.4)	20.5 (11.9)
	2	25 (15.9)	25.4 (15.8)	24.1 (14.7)	24.1 (14.7)
	4	36 (15.7)	35 (15.5)	36.9 (18.8)	36.5 (18.6)
	6	40 (17.9)	44.8 (18.7)	41.6 (20.5)	45.5 (19.6)
	8	49 (24.8)	50.1 (23.8)	50.7 (23.5)	51.2 (23.0)

### Specific measures of tinnitus perception

**FIGURE 3:**
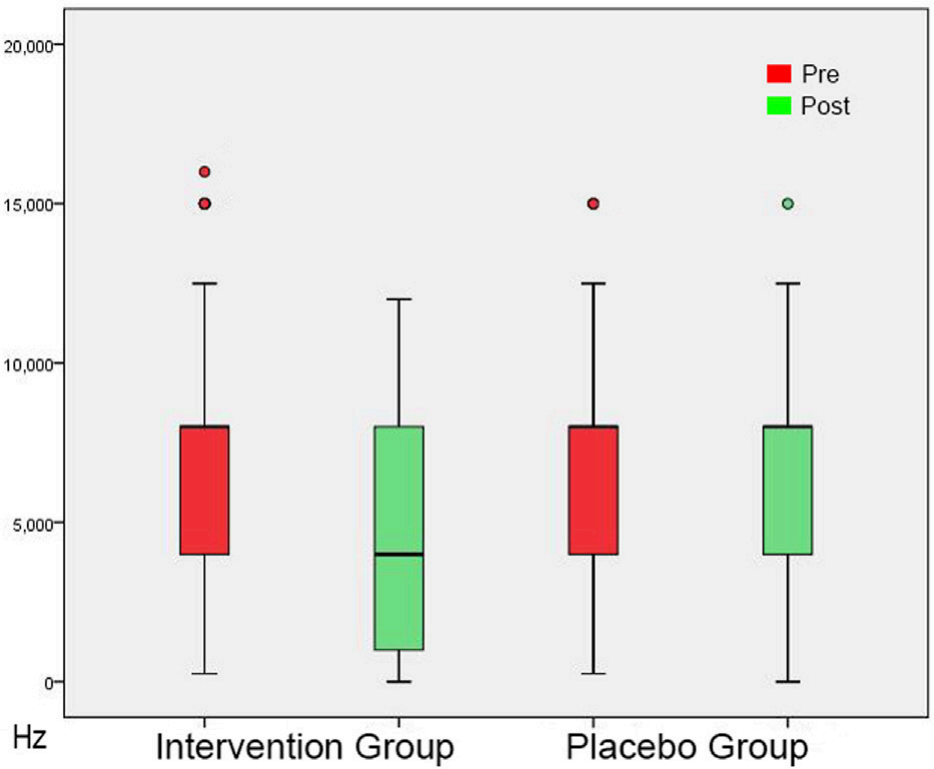
Tinnitus Pitch shown as box plots for each group in initial session and in post-3-month session. The line inside the box represents the median and the box is defined by two lines at the First Quartile, the value below which 25% of the values in the data set are found, and the Third Quartile, the value below which 75% of the values in the data set are found. The whiskers extend to the most extreme data points not considered outliers, and the outliers are plotted individually as circles. From this Figure it is apparent that the Tinnitus Pitch data are skewed towards the higher frequencies.

**FIGURE 4:**
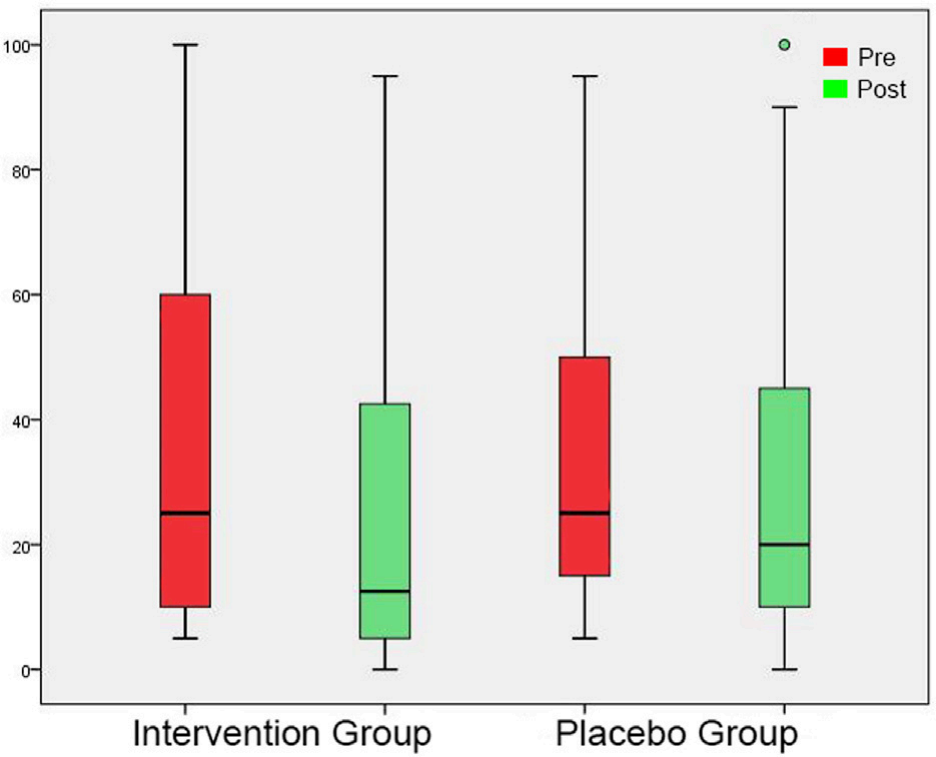
Tinnitus Loudness at the perceived tinnitus pitch shown as box plots for each group in initial session and in post-3-month session. On each box, the line inside the box indicates the median, and the bottom and top edges of the box indicate the 25th and 75th percentiles, respectively. The whiskers extend to the most extreme data points not considered outliers, and the circles indicate outliers.

**FIGURE 5:**
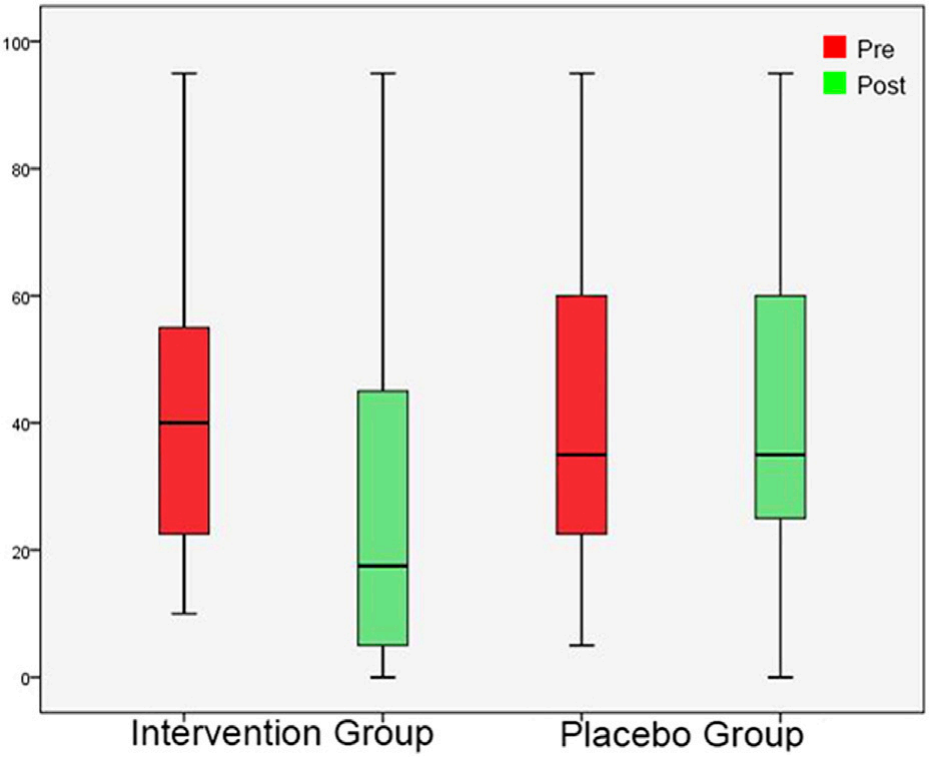
Minimum Masking Level shown as box plots for each group in initial session and in post-3-month session. On each box, the line inside the box indicates the median, and the bottom and top edges of the box indicate the 25th and 75th percentiles, respectively. The whiskers extend to the most extreme data points not considered outliers.

In the initial session, tinnitus pitch, loudness at tinnitus pitch, loudness at 1 kHz, MML and RI were similar in both groups (Tables 4, 5). In the post-3-month session (Tables 4, 5), there were significantly lower tinnitus pitch (Figure 3), loudness at tinnitus pitch (Figure 4), loudness at 1 kHz, MML (Figure 5) and RI values in the intervention group. In both groups, tinnitus pitch, loudness at tinnitus pitch and loudness at 1 kHz diminished significantly throughout the follow-up period, but the degree of decrease was significantly greater in the intervention group. Regarding MML, it decreased significantly in repeat session compared to the initial one only in the study group and subsequently the degree of decrease differed significantly between the two groups.

**TABLE 4:** Mean values and standard deviation (SD) of tinnitus pitch, loudness at tinnitus pitch, loudness at 1 kHz, MML, THI and Mini-TQ by Group and Session.

	Group	Initial session	Post-3-month session	p2	p3
		Mean (SD)	Mean (SD)		
Tinnitus pitch (Hz)	Placebo	7207.5 (3264.5)	6065 (3418.4)	<0.001	0.002
	Intervention	7574.5 (3639.0)	4607 (3497.9)	<0.001	
	p1	0.497	0.004		
Loudness at tinnitus pitch	Placebo	34.0 (26.3)	31.1 (26.2)	<0.001	<0.001
	Intervention	33.6 (27.5)	24.8 (27.4)	<0.001	
	p1	0.660	0.008		
Loudness at 1 kHz	Placebo	24.4 (21.9)	22.5 (21.4)	0.005	<0.001
	Intervention	24.3 (22.8)	19.0 (22.7)	<0.001	
	p1	0.892	0.029		
MML	Placebo	41.2 (22.8)	39.9 (24.2)	0.236	<0.001
	Intervention	43.6 (23.4)	28.7 (25.6)	<0.001	
	p1	0.487	0.001		
THI	Placebo	32.3 (17.9)	28.4 (18.4)	<0.001	<0.001
	Intervention	33 (22.3)	21.4 (21.9)	<0.001	
	p1	0.604	0.001		
Mini-TQ	Placebo	9.3 (4.2)	7.9 (5.0)	<0.001	<0.001
	Intervention	10.0 (5.0)	6.1 (5.3)	<0.001	
	p1	0.523	0.001		

*p* ^*1*^-value for group effect (via Mann-Whitney test); *p* ^*2*^-value for time effect (via Wilcoxon sign test); *p* ^*3*^-value for time*group (via repeated measures ANOVA, with logarithmically transformed data).

**TABLE 5:** Residual Inhibition by group and session.

Residual inhibition	Placebo group		Intervention group		*p* ^*2*^	*p* ^*3*^
	Pre	Post	Pre	Post		
Absent, N (%)	14 (14)	11 (11)	13 (12.5)	6 (5.8)	0.774	0.007
Complete, N (%)	56 (56)	54 (54)	55 (52.9)	78 (75)		
Partial, N (%)	30 (30)	35 (35)	36 (34.6)	20 (19.2)		
p1	0.174		<0.001			

*p* ^*1*^-value for time effect (via McNemar test); *p*_*2*_-value for group effect in pre-measurements (Pearson’s chi-square test); *p* ^*3*^-value for group effect in post measurements (Pearson’s chi-square test).

No significant differences in RI were found between the two groups in the initial measurement. In the post-3-month measurement, there was a significant difference between the two groups and more specifically the percentage of C was greater in the intervention group. In the placebo group, there were no significant differences between the two measurements. On the contrary, in the intervention group, there was a significant increase of C percentage in the post-3-month measurement compared to the initial one. RI by group and measurement is presented in Table 5.

### Questionnaires

**FIGURE 6:**
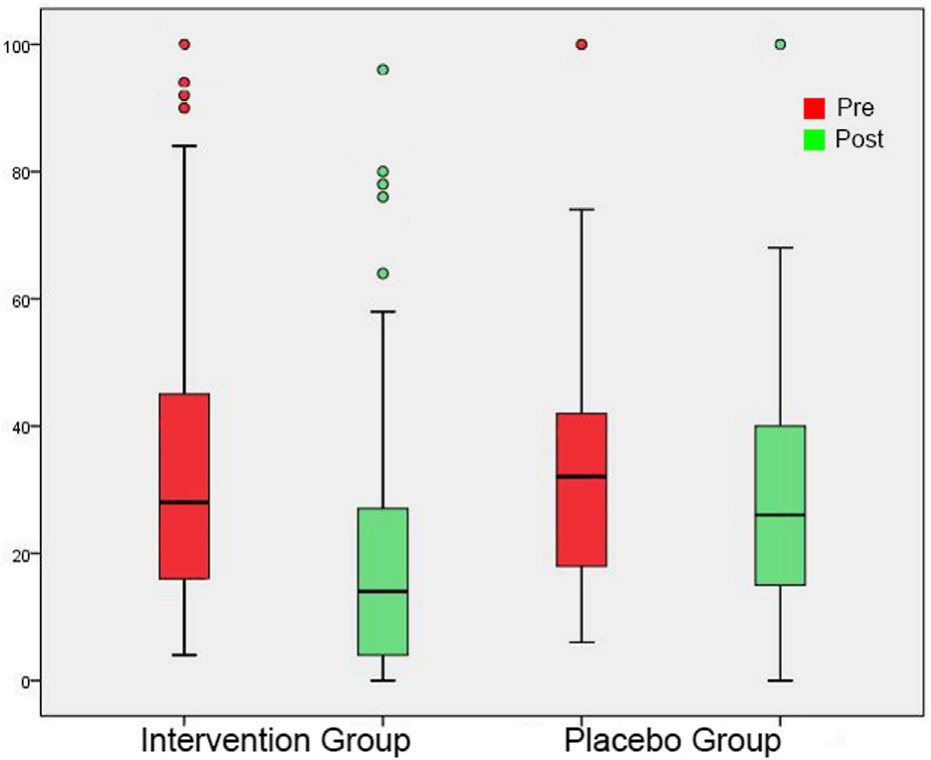
THI shown as box plots for each group in initial session and in post-3-month session. On each box, the line inside the box indicates the median, and the bottom and top edges of the box indicate the 25th and 75th percentiles, respectively. The whiskers extend to the most extreme data points not considered outliers, and the circles indicate outliers.

**FIGURE 7:**
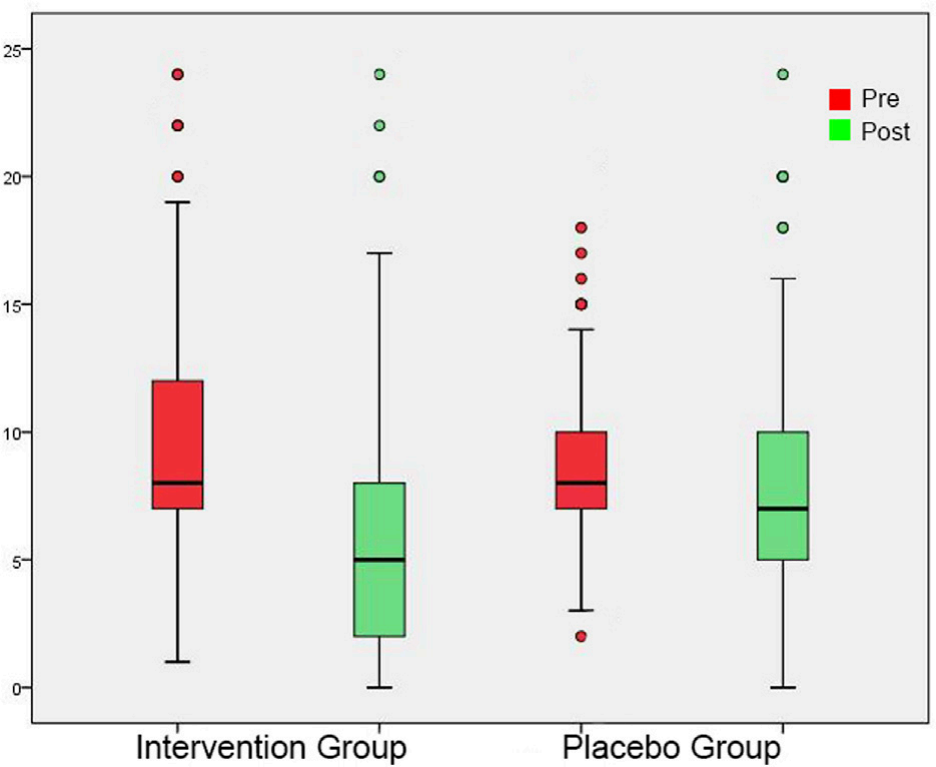
Mini-TQ shown as box plots for each group in initial session and in post-3-month session. On each box, the line inside the box indicates the median, and the bottom and top edges of the box indicate the 25th and 75th percentiles, respectively. The whiskers extend to the most extreme data points not considered outliers, and the circles indicate outliers.

In the initial measurement, THI and Mini-TQ were similar in both groups (Tables 4, 5). At the post-3-month measurement, there were significantly lower THI and Mini-TQ values in the intervention group. In both groups, THI and Mini-TQ diminished significantly throughout the follow-up period, but the degree of decrease was significantly greater in the intervention group (Figures 6, 7). Comparison of the two subgroups with duration 0-6 months and 7-12 months in the intervention group, showed lower THI and Mini-TQ values after treatment in the 0-6 months subgroup, which were marginally statistically significant (THI: *p* = 0.042; Mini-TQ: p-0.029), implying probably a small beneficial effect of spontaneous remission. However, in the placebo group, THI and Mini-TQ values after treatment did not differ significantly between the two subgroups (THI: *p* = 0.783; Mini-TQ: p-0.893).

**FIGURE 8:**
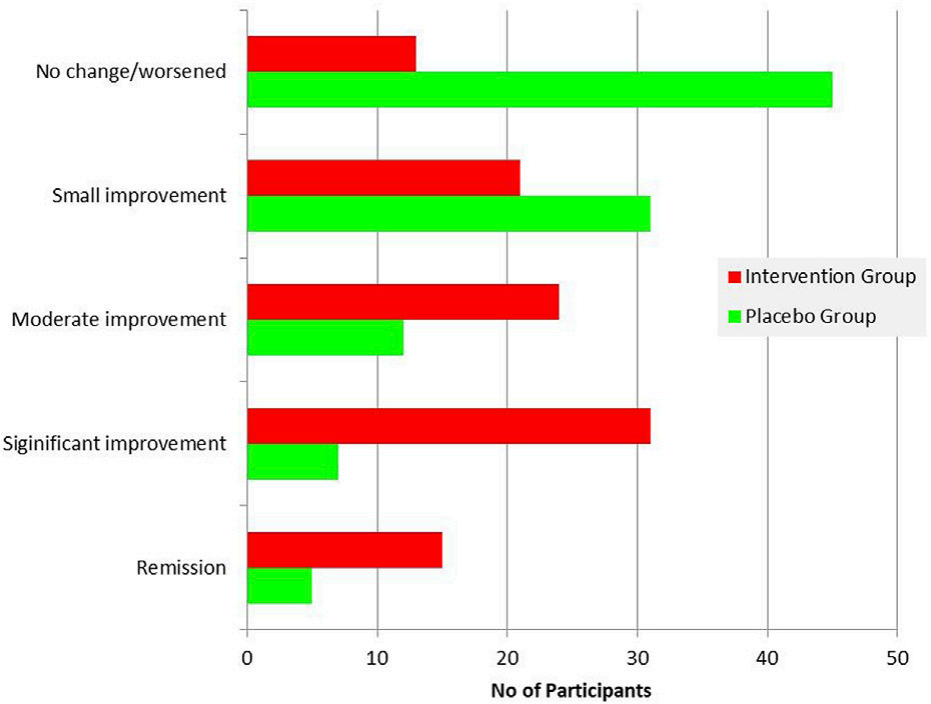
Patients’ Global Impression of Change (PGIC) response summary for each group.

The PGIC score differed significantly between the two groups (Table 6), with the improvement being significantly greater in the patients’ group (Figure 8). Comparison of the two subgroups with duration 0-6 months and 7-12 months showed significant difference of PGIC score between the two groups (chi square = 13.956, *p* = 0.007) at the intervention group, but absence of statistically significant at the placebo group (chi square = 5.243, *p* = 0.263).

**TABLE 6:** Patients’ Global Impression of Change (PGIC) response for each group.

	Placebo group	Intervention group	*p* ^*1*^
PGIC	N (%)	N (%)	
No improvement/worsened	45 (45)	13 (12.5)	<0.001
Small improvement	31 (31)	21 (20.2)	
Moderate improvement	12 (12)	24 (23.1)	
Significant improvement	7 (7)	31 (29.8)	
Tinnitus remission	5 (5)	15 (14.4)	

*p* ^*1*^-value from Pearson’s chi-square test.

## Discussion

Our randomized, double-blind, placebo-controlled, prospective study was carefully designed to assess the effect of the use of MemoVigor 2, a food supplement that combines several therapeutic ingredients, in a group of patients with idiopathic tinnitus divided into an intervention group and a placebo group. To evaluate changes in tinnitus we used audiometric measures, specific measures of tinnitus perception and tinnitus questionnaires. Audiometric results were similar in the two groups, both in the initial testing session and in the post-3-month session, probably because of the short time that elapsed between the two measurements. We found that all tinnitus measures, including tinnitus pitch, loudness at tinnitus pitch, loudness at 1 kHz, MML and RI, improved significantly in the intervention group. Most of these measures improved in the placebo group too, but to a lesser degree. MML and RI improved only in the intervention group and not in the placebo group. To evaluate tinnitus-related discomfort we used two tinnitus questionnaires, the widely used THI which measures the impact of tinnitus on patient’s quality of life, and the Mini-TQ, a reliable tool for rapid and reliable assessment of subjective tinnitus distress. Both questionnaire scores diminished significantly in both groups, but the degree of decrease was greater in the intervention group. Finally, the participants reported the tinnitus outcome after treatment using the PGIC score which differed significantly between the two groups, with greater improvement observed in the intervention group.

We selected patients with subjective tinnitus of recent onset ≤12 months. According to the Clinical Practice Guideline for Tinnitus of the American Academy of Otolaryngology-Head and Neck Surgery (Tunkel et al., 2014), the period to distinguish between recent onset and persistent tinnitus is 6 months. However, we studied patients with tinnitus duration up to 12 months, to minimize the phenomenon of spontaneous improvement or resolution that is common in sufferers within the first 6 months of the disease, so that we could evaluate if there is a certain therapeutic value of the food supplement studied. Additionally, research data exist (Carpenter-Thompson et al., 2015) that there are functional differences in neural correlates of tinnitus in adults, based on the amount of time for which they have experienced tinnitus. Patients with tinnitus lasting less than 1 year associate posterior cingulate and insula with emotional reaction to tinnitus, whereas patients having tinnitus longer than 1 year recruit more frontal regions of the brain to better control their emotional response. We did not study patients with persistent tinnitus over 1 year, because many psychological aspects interfere and some studies suggest that tinnitus may worsen depression, generalized anxiety, and the quality of life, while others have found that tinnitus improves over time spontaneously and there appears to be habituation in a sizeable percentage of patients over a prolonged period (Phillips et al., 2018; Han et al., 2021). Accordingly, the therapeutic value of MemoVigor 2 might be not quite clear, since various psychological and social aspects of tinnitus can severely affect the patients.

To evaluate a possible effect of spontaneous remission of tinnitus in our subjects we divided the intervention and the control group into two subgroups, with tinnitus duration 0-6 months and 7-12 months, and compared their results. In the intervention group, THI and Mini-TQ scores were marginally lower in the first group, implying a possible effect of spontaneous improvement during the first 6 months to several patients. In the two subgroups of the placebo-treated subjects, mean values were somewhat lower in the first group but did not reach statistical significance. We found similar results in the evaluation of the PGIC score. Accordingly, the role of tinnitus spontaneous remission in our subjects is not quite clear but is probably small. We can also hypothesize that MemoVigor 2 is more effective if administered during the first months of the disease, since later various psychological factors could interfere negatively.

In the diagnostic evaluation of tinnitus, we used both specific measures of tinnitus perception, such as tinnitus pitch and loudness, and questionnaires, since the overall impact of tinnitus is not only influenced by the characteristics of the tinnitus but also by the psychological reactions of each tinnitus sufferer. Both sets of testing are necessary and complementary because the perceptual aspects of tinnitus do not always explain the subjectively perceived severity of the symptom (Langguth et al., 2019). Tinnitus questionnaires are appropriate tools for evaluating the patient’s reaction to tinnitus. We selected the THI and the Mini-TQ because both are standardized, reliable and have been widely used, having also validated versions in the Greek language (Panagiotopoulos et al., 2015; Papitsi et al., 2020).

Among several treatment modalities which have been implemented in patients with tinnitus, pharmacotherapy has a prominent place. It seems that tinnitus can be pharmacologically assessed and treated as evidenced by the transient dose-dependent reduction of tinnitus in up to 70% of patients after intravenous application of lidocaine (Israel et al., 1982; Kleinjung and Langguth, 2021). Unfortunately, lidocaine is only effective when applied intravenously and its effect is short-lasting. Although there are no specific pharmacological compounds that have been approved for the treatment of chronic tinnitus, a large variety of drugs approved for other indications have been investigated for the treatment of tinnitus, mostly with little evidence of greater benefit than harm. These drugs include various antianxiety, anaesthetics, antiarrhythmics, anticonvulsants, antidepressants, prostaglandins, antihistamines, muscle relaxants, calcium channel blockers, diuretics, glutamate receptor antagonists, melatonin, and others (Kim et al., 2021).

The main reason that a drug for the treatment of tinnitus is not effective in all patients is that tinnitus itself cannot be considered as a disease but rather as a symptom. Tinnitus is a heterogeneous and complex condition with multiple associated biological, psychological and contextual contributors, which are difficult to diagnose and treat (Procházková et al., 2018; Trochidis et al., 2021). The aetiology might be otological, neurological, cardiovascular, endocrinological, metabolic, musculoskeletal or mental and the pharmacological therapeutic approach should take the suspected possible cause in each case into consideration. The use of a food supplement such as MemoVigor 2 which contains a combination of therapeutic substances has the advantage that it may be successful in several categories of patients with tinnitus since a combination of substances interfering with multiple neurotransmitter systems might result in better effects than substances acting on a single receptor.

MemoVigor 2 is a dietary supplement that contains a combination of ingredients, such as dry extract from G. biloba leaves and Bilberry fruits, phospholipids, L-acetylcarnitine, vitamins B, E and C, as well as trace minerals, such as selenium, magnesium and potassium. Most of them are antioxidants that have been previously used for tinnitus, and also for the treatment of other peripheral and central vestibular syndromes, and of lesions of the cochlear and the facial nerve.

Antioxidants are substances that act against the effects of oxidative stress, triggered by oxidizing compounds and free radicals, such as reactive oxygen species (ROS) and reactive nitrogen species (RNS) (Fujimoto and Yamasoba, 2014). Since oxidative stress seems to be involved in the pathogenesis and progression of several diseases, antioxidants are important because they inhibit damage to DNA and to macromolecules, thus alleviating the cumulative damage that can trigger these diseases. Cochleae with the high metabolic demands of their mechanosensory hair cells are extremely vulnerable to oxidative stress because of the damaging effects of mitochondrial ROS (Gonzalez-Gonzalez, 2017). Elevated ROS concentration leads to genetic and cellular alterations and induces impaired blood flow to the cochlea, fused hair cell stereocilia and degeneration of the stria vascularis, the supporting structures and nerve fibers. These changes result in permanent cochlear degeneration and apoptosis.

In relation to tinnitus, there are reports of higher plasma concentrations of oxidative stress biomarkers and low antioxidant activity, supporting the role of oxidative status in the pathogenesis of tinnitus (Koç et al., 2016; Celik and Koyuncu, 2018). Antioxidant therapy in patients with idiopathic tinnitus could reduce oxidative stress and damage to the inner ear, also reducing tinnitus loudness and discomfort. However, research data on the efficacy of antioxidant supplementation in tinnitus are scarce and conflicting, although there are some recent reports that show tinnitus improvement after treatment with antioxidants (Procházková et al., 2018; Oppitz et al., 2022). Additionally, emerging evidence suggests that neuroinflammation may play a large role in the development of tinnitus and the influence of various proinflammatory cytokines such as TNF-α, IL-6, β-2GP1 and IL-1 has been described. Neuroinflammation is the central nervous system’s response to potentially harmful stimuli such as injury, infection, disease, or abnormal neural activity. It was demonstrated that hearing loss or noise exposure causes chronic neuroinflammation in the peripheral and central auditory pathways. Consequently, noise trauma or hearing loss-related disorders might trigger a neuroinflammatory response in which pro-inflammatory cytokines influence synaptic transmission that results in an excitation–inhibition imbalance, speculated to be the mechanism of tinnitus (Adcock and Vanneste, 2022). Anti-TNF-α therapy, such as Etanercept, was studied to prevent noise-induced hearing loss (Dhukhwa et al., 2019), and was recently studied in tinnitus as well (Shulman et al., 2021).

Extracts from the leaves of the plant G. biloba, which is native to China and East Asia in general, have been used for many years in various neurological and other disorders, such as dementia, cognitive disorders, headaches, dizziness, mood disorders, cardiovascular diseases and coronary heart disease (Liu et al., 2023). G. biloba extracts have also been used for the treatment of tinnitus. Its major metabolites are flavonoids and terpenoids, and it has been proven that it possesses antioxidant activity, increases tolerance to hypoxia, improves blood flow, increases the flexibility of the cellular elements of blood and improves microcirculation. It also affects the levels of neurotransmitters, increases neuroplasticity, provides neuroprotection and prevents cerebral edema (Luetzenberg et al., 2020; Liu et al., 2023). Such action mechanisms may help in the treatment of tinnitus, reducing damage caused by free radicals to the cochlea, or by increasing the blood flow of the inner ear.

Various studies as well as several systematic reviews have been conducted for the usefulness of G. biloba in the treatment of tinnitus, but the existing evidence from these studies remains inconclusive for the effectiveness of G. biloba extract. Han et al. (Han et al., 2012) studied a group of 38 patients with tinnitus in a randomized crossover study. Patients received G. biloba EGB 761 extract, or clonazepam for the first 3 weeks and switched to the other drug after a 2-week washout when no medication was taken. For the final 3 weeks, subjects were instructed to increase the dose until they perceived a satisfactory improvement in their tinnitus or intolerable side effects. The authors found that clonazepam was effective but EGB 761 had no significant effect on tinnitus. Polanski et al. (2016) studied in a randomized, double-blinded, placebo-controlled trial 58 elderly subjects with tinnitus and associated sensorineural hearing loss treated with G. biloba dry extract (120 mg/day) and several other antioxidants and did not find any benefit from the treatment. In a recent Cochrane Database review of 12 studies with 1915 participants (Sereda et al., 2022), the authors concluded that there is uncertainty about the benefits and harms of G. biloba for the treatment of tinnitus when compared to placebo.

On the other hand, several studies have found positive results in the use of G. biloba for the treatment of tinnitus. Procházková et al. (Procházková et al., 2018) in a recent double-blind randomized trial of 200 subjects with sub-chronic or chronic tinnitus who received either G. biloba Egb 761 extract or pentoxifylline over 12 weeks found significant improvement in all tinnitus questionnaires used. Nishad et al. (Nishad et al., 2019) evaluated the effect of treatment with a single intravenous injection of caroverine and G. biloba extract (60 mg twice a day) in 86 patients with tinnitus. They found that a single dose infusion of caroverine immediately improved tinnitus in 54.5% of the patients, but the improvement was not sustained at a 3-6 months follow-up. G. biloba was also effective in improving tinnitus in 31.8% of cases and improvement was sustained even after 3 months of cessation of treatment. The authors concluded that G. biloba should be used for 3 months to provide sustained and long-lasting relief.

Von Boetticher (von Boetticher, 2011), in a review of double-blind studies with placebo control, found that only 3 of the numerous reports that studied the efficacy of G. biloba in the treatment of tinnitus fulfilled the necessary criteria for inclusion in the review, and 5 more that studied efficiency not only in tinnitus but also in other agnosia disorders and dementia. According to the author, all 8 studies showed statistically significant superiority of active treatment *versus* placebo. Therefore, he concluded that the extracts of G. biloba are suitable for the treatment of tinnitus, as either a single symptom or in combination with dementia or age-related cognitive disorders.

Carnitine, another ingredient of MemoVigor 2, has a strong antioxidant activity and possesses a key role in mammalian lipid metabolism. It is mainly required in the transport of activated fatty acids from the cytosol into the mitochondrial matrix for subsequent oxidation and it may enhance the mitochondrial bioenergy and biogenesis (Berni et al., 2008). L-Carnitine, a form of carnitine used pharmacologically, acts as a scavenger of reactive oxygen species, a possible cause of tinnitus in patients (Wu et al., 2011). Τhis is the reason why L-carnitine has been successfully used in the treatment of tinnitus (Gopal et al., 2015).

MemoVigor 2 contains vitamins of groups B, E and C. Vitamins of groups E and C are known for their antioxidant action. Savastano et al. (Savastano et al., 2007) treated 31 patients with tinnitus with glycerophosphorylcholine, glycerophosphorylethanolamine, b-carotene and vitamins C and E for three courses of 6 weeks with a period of 2 weeks between each course. The patients showed a significant reduction in their tinnitus measured using VAS and tinnitus loudness. The complex of vitamin B is a family of vitamins that have been grouped because of their interaction with the functions of the human enzyme systems, and because of their allocation in natural food sources. A deficiency of vitamin B complex, specifically B12, was associated with tinnitus, while intake may improve symptoms (Shemesh et al., 1993; Singh et al., 2016). Α mechanism implicated in Vitamin B12 deficiency neuropathy is hypomethylation in the central nervous system. Cochlear function is dependent on the normal functioning of nerve tissue and the existence of adequate vascular supply. Vitamin B12 deficiency may cause the demyelination of neurons in the cochlear nerve, resulting in hearing loss. Additionally, low levels of Vitamin B12 is associated with the destruction of the microvasculature of the stria vascularis, which might result in decreased endocochlear potential and in hearing loss and tinnitus (Krumholz et al., 1981).

MemoVigor 2 contains also trace minerals: magnesium, potassium and selenium. Magnesium is essential for numerous cellular processes including enzymatic reactions, metabolic cycles, cellular signaling, DNA/RNA stabilities, and serves as a major regulator of calcium channels involved in neurotransmission (Yamanaka et al., 2019). Therefore, magnesium plays a significant role in neural and central auditory pathways. Oral magnesium therapy was reported to be effective in the prevention of cochlear damage in noise-induced hearing loss because magnesium prevents apoptosis in hair cells by reducing calcium flow into the cell and reduces ischemia by causing vasodilatation in the cochlear arterioles (Attias et al., 2004; Abaamrane et al., 2009). It has also been used successfully in the treatment of tinnitus (Cevette et al., 2011).

Potassium is the major electrolyte in the intracellular fluid. Potassium channels regulate excitability in neuronal, sensory and muscular cells and they control cell volume, proliferation, differentiation and survival. In mice, it has been shown that tinnitus generation after noise exposure is related to increased spontaneous firing of fusiform cells within the dorsal cochlear nucleus (Li et al., 2013). This hyperactivity is caused by decreased potassium currents mainly through the Kv7 channels. Although the potential involvement of potassium channels in tinnitus pathophysiology has been the subject of several studies (Li et al., 2015; Langguth et al., 2016), the effect of drugs that act on Kv7 channels on tinnitus, such as flupirtine and retigabine has not been adequately investigated. In addition to Kv7, Pilati et al. (2012) showed that the reduction in Kv3 channel activity underlies increased fusiform cell bursting as well. Together with increased excitatory neurotransmission and decreased inhibitory neurotransmission, changes in intrinsic membrane excitability within the dorsal cochlear nucleus are associated with the development of tinnitus. However, a clinical trial of AUT00063, an experimental new medicine that has been demonstrated to suppress spontaneous hyperactivity by modulating the action of Kv3 voltage-gated potassium channels in central auditory cortical neurons of a rodent model, showed that AUT00063 was not effective in alleviating tinnitus symptoms in humans (Hall et al., 2019).

Selenium is associated with the activity of glutathione peroxidase, which is a cochlear anti-oxidant enzyme that deactivates ROS and RNS and specifically detoxifies H2O2 which induces ciliary dysfunction and apoptosis of hairy cells of the inner ear (Atila et al., 2021). A deficiency of selenium reduces the effectiveness of glutathione peroxidase increasing H2O2 level and oxidative stress, and for this reason, it has been used in combination with B and E vitamin complexes in the treatment of idiopathic sudden hearing loss (Kaya et al., 2015).

Finally, phospholipids are contained in MemoVigor 2. These are a large class of fatlike, phosphorus-containing metabolites that participate in the structure of cell membranes and are oxidized by free radicals in OH or COH thus compromising the membrane integrity. Therefore, phospholipid sufficiency is essential, because they are membrane stabilizers (Dai et al., 2021). Patients with idiopathic tinnitus may have high levels of ROS in their blood samples (Neri et al., 2002). Oxidative stressors induce the production of ROS, which interact with the phospholipidic membrane of the sensory cells producing aldehyde lipids such as the 4-hydroxynonenal, a mediator of apoptosis for auditory neurons and hair cells and it has been found that the use of phospholipids in the treatment of tinnitus may have favourable results (Savastano et al., 2007).

The administered food supplement in this study, which contains a combination of the previously described ingredients with antioxidant, vasoactive and neurogenic properties, had a positive outcome in our intervention group. It appears that treatment with antioxidants in patients with idiopathic tinnitus reduces oxidative stress which harms the inner ear tissues, whereas vasoactive and neurogenic properties of the included ingredients improve circulation and structural integrity of neural circuits. Moreover, we have found that patient tolerance towards tinnitus improves. It should be noticed that tinnitus in the placebo group patients has also improved, although in a lesser degree in comparison with the intervention group. This agrees with the results of a well-designed systematic review and meta-analysis of controlled trials assessing tinnitus interventions (Phillips et al., 2018). The authors of this review concluded that patients demonstrate a small but statistically significant improvement in self-reported global tinnitus severity scores over time, despite receiving no intervention, a finding that provides statistical evidence that tinnitus generally improves over time, albeit the effect is highly variable across individuals.

The large sample size, the high treatment adherence and the application of a complete set of tinnitus evaluation tests including audiometric measures, specific measures of tinnitus perception and tinnitus questionnaires, may be considered as strengths of this trial. Limitations of the study are the single-centre setting and the inclusion exclusively of patients with recent onset tinnitus. Further randomized double-blind placebo-controlled trials are needed to investigate the effectiveness of MemoVigor 2 in the treatment of persistent tinnitus.

## Conclusion

We showed that the use of a therapeutic supplement, which contained phospholipids, L-acetylcarnitine, G. biloba and Bilberry dry extracts, several vitamins and trace minerals, improved recent-onset tinnitus, as proved by a set of tests performed for its evaluation, including audiometric measures, specific measures of tinnitus perception and tinnitus questionnaires. Tinnitus in the placebo group improved too but to a lesser degree.

## Data Availability

The datasets presented in this study can be found in online repositories. The names of the repository/repositories and accession number(s) can be found below: https://figshare.com/articles/dataset/RawData-MemoVigor-2-DB_xlsx/23614896 Online Data Repository “Figshare”.
