# Correction: The effect of MemoVigor 2 on recent-onset idiopathic tinnitus: a randomized double-blind placebo-controlled clinical trial

**DOI:** 10.3389/fphar.2026.1856536

**Published:** 2026-05-07

**Authors:** Dimitrios G. Balatsouras, Isidora Papitsi, George Koukoutsis, Michael Katotomichelakis

**Affiliations:** 1 Department of Otorhinolaryngology, Tzaneio General Hospital, Piraeus, Greece; 2 Department of Otorhinolaryngology, Medical School, Democritus University of Thrace, Komotini, Greece

**Keywords:** tinnitus, antioxidants, placebo, oxidative stress, diet supplement, pharmacotherapy, clinical trials, hearing loss

Bionat is incorrectly referred to as the manufacturer, whereas it is in fact the distributor in Greece. The correct manufacturing company is EUROPHARMA. The acknowledgements statement was erroneously given as “The authors are thankful to Bionat for the kind donation of supplements and placebo pills. The authors are also thankful to the statistician Chara Tzavara for her assistance in statistical analysis.” The correct statement is “The authors are thankful to EUROPHARMA for the kind donation of supplements and placebo pills. The authors are also thankful to the statistician Chara Tzavara for her assistance in statistical analysis.”

The manufacturer was erroneously given as Bionat.

A correction has been made to the section Materials and Methods, *Randomization and blinding process*, Paragraph Number 1:

“All consecutive patients with subjective tinnitus examined in our |Clinic, who fulfilled the inclusion criteria, participated in the study. The investigators informed the eligible patients regarding the aims, methods, anticipated benefits and potential hazards of the study, and provided them with an information sheet. Informed consent was obtained from each patient who agreed to participate in the study. Subjects were randomly allocated to take either one tablet of 900 mg of MemoVigor 2, or a placebo tablet daily for 3 months, in a 1:1 ratio. MemoVigor 2 and placebo tablets were identical in colour, weight and size, and were both provided by the manufacturing company of MemoVigor 2, EUROPHARMA, placed inside bottles with identical appearance, with each bottle containing tablets for a 3-month treatment. A completely randomized design was used for both treatments using the Number Generator of the Research Randomizer (www.randomizer.org). Two sets of numbers were created and an independent contributor not involved in the study, assigned each number to one bottle. The independent contributor gave each patient a bottle, recorded the randomization number corresponding to each patient and created a list matching drug numbers with treatments, which was unavailable to investigators. Only the independent contributor had access to the group assignments and neither the patients nor the investigators were aware of the treatment allocation.”

“Memovigor 2 (Bionat, Athens, Greece)” incorrectly identifies Bionat as the manufacturing company; however, Bionat is the distributor in Greece, whereas the manufacturing company is EUROPHARMA.

A correction has been made to the section Materials and Methods, *Intervention,* Paragraph Number 1:

“Randomized patients were given either one tablet 900 mg of MemoVigor 2 (EUROPHARMA, Torino, Italy) or a placebo, daily. MemoVigor 2 is a commercial product with batch number EUPH 0034 M10550_11/23 that contains various ingredients, such as phospholipids, L-acetylcarnitine, vitamins B1, B6, B12, C, and E, Ginkgo biloba and Bilberry plant extracts, as well as trace minerals, including selenium, magnesium and potassium. The full list of ingredients with the amount per tablet is shown in Table 1. Ginkgo biloba extract is 3% dry extract prepared from the leaves of the plant Ginkgo biloba L. [Ginkgoaceae], extraction solvent: ethanol and excipient: maltodextrin 35%. It contains ≥3% flavonol glycosides and ≤ 5ppm ginkgolic acids and is prepared from Suisse Nutraingredients, Monza, Italy. Bilberry extract is 1% dry extract prepared from the fruits of the plant Vaccinium myrtillus L. [Ericaceae], extraction solvent: water and excipient: maltodextrin 35%. It contains ≥1% anthocyanins and is prepared from Fagron Italia, Bologna, Italy. The intervention lasted 3 months and the patients were instructed to continue their usual medical treatment, diet, and exercise habits during this period and were advised to record and report any adverse reaction. Patients were recruited between December 2019 and March 2023.”

The original article has been updated.

